# Complex patterns of response to oral hygiene instructions: longitudinal evaluation of periodontal patients

**DOI:** 10.1186/s12903-018-0537-z

**Published:** 2018-05-02

**Authors:** Felice Amoo-Achampong, David E. Vitunac, Kathleen Deeley, Adriana Modesto, Alexandre R. Vieira

**Affiliations:** 1 Departments of Oral Biology, University of Pittsburgh School of Dental Medicine, 412 Salk Pavilion, Pittsburgh, PA 15261 USA; 2 Pediatric Dentistry, University of Pittsburgh School of Dental Medicine, 412 Salk Pavilion, Pittsburgh, PA 15261 USA

**Keywords:** Periodontitis, Dental plaque, Oral biofilm, Oral hygiene

## Abstract

**Background:**

Oral hygiene instruction is an intervention widely practiced but increased knowledge about oral health does not necessarily dramatically impact oral disease prevalence in populations. We aimed to measure plaque and bleeding in periodontal patients over time to determine patterns of patient response to oral hygiene instructions.

**Methods:**

Longitudinal plaque and bleeding index data were evaluated in 227 periodontal patients to determine the impact of oral hygiene instructions. Over multiple visits, we determined relative plaque accumulation and gingival bleeding for each patient. Subsequently, we grouped them in three types of oral hygiene status in response to initial instructions, using the longitudinal data over the period they were treated and followed for their periodontal needs. These patterns of oral hygiene based on the plaque and gingival bleeding indexes were evaluated based on age, sex, ethnic background, interleukin 1 alpha and beta genotypes, diabetes status, smoking habits, and other concomitant diseases. Chi-square and Fisher’s exact tests were used to determine if any differences between these variables were statistically significant with alpha set at 0.05.

**Results:**

Three patterns in response to oral hygiene instructions emerged. Plaque and gingival bleeding indexes improved, worsened, or fluctuated over time in the periodontal patients studied. Out of all the confounders considered, only ethnic background showed statistically significant differences. White individuals more often than other ethnic groups fluctuated in regards to oral hygiene quality after instructions.

**Conclusions:**

There are different responses to professional oral hygiene instructions. These responses may be related to ethnicity.

**Electronic supplementary material:**

The online version of this article (10.1186/s12903-018-0537-z) contains supplementary material, which is available to authorized users.

## Background

The two most common oral diseases (dental caries and periodontitis) are bacteria-mediated and can be prevented by satisfactory dental biofilm control. Dental biofilm starts forming soon after its removal from the tooth surface meaning that effective dental biofilm control requires effectively disturbing biofilm formation on a daily basis.

Epidemiology of dental caries and periodontitis in many developed countries has changed in the last five decades due to improved oral health practices. These changes also emphasize disparities in oral health, with most oral disease burden affecting the socially disadvantaged individuals [1].

A paradigm shift has occurred in dentistry where the emphasis has changed from it being a ‘repair service’ to being a health care service that prevents disease prior to damage occurring. Since the 1990’s, oral health promotion has been considered of high importance to the service that dentists provide to their patients [2].

The implementation of oral health promotion has two aspects: the provider and the individual benefiting from this practice. On one hand, the dentist is the agent providing the intervention, and on the other hand is the patient, who will benefit from effectively implementing daily good oral health practices. In the United States, the dentist can be reimbursed by performing professional dental prophylaxis, which includes scaling and polishing procedures to remove coronal plaque, calculus, and stains (dental code D1110-Prophylaxis-Adult). This code also implies the provision of oral hygiene instructions, since dental code D1330 can be used in cases where additional time and expertise is directed toward the client’s care beyond that of the routine brushing and flossing instructions included in the prophylaxis procedure codes, such as D1110. Regarding the patient, it is suggested that the psychology of behavior change is key to successful oral health promotion [3].

The two simplest and most widely used ways to promote oral health is through verbal and written advice. These two approaches are effective for increasing patient knowledge regarding oral health (reviewed in [3]) but data suggest that this does not have an impact on oral disease presentation. In our study, we took advantage of longitudinal data to determine how oral health instructions provided by a dentist impact oral health practices as measured by presence of dental biofilm and gingival bleeding in follow up dental visits. These direct measurements of oral hygiene practices are better surrogates than the traditional self-reported data of the number of times and when tooth brushing and flossing are performed every day.

## Methods

### Sample population

The subjects of this study were patients with active periodontitis being treated at the Department of Periodontics and Preventive Dentistry at the University of Pittsburgh’s School of Dental Medicine. All of them were being treated by scaling and root planning and being monitored regarding personal oral hygiene improvements. Individual samples and clinical information were obtained through the Dental Registry and DNA Repository project. Since 2006, this project seeks to provide all patients seeking treatment at the School of Dental Medicine with the opportunity to be a part of the registry. All subjects in this study were selected from this database with a focus on those who have received periodontal treatment through the School of Dental Medicine. The information consisted of diagnostics, treatment planning and treatments provided as stated in each subject’s record. All participants signed a consent form that authorized the use of information from their dental records and provided a saliva sample as a source of genomic DNA (deoxyribonucleic acid). Extracted DNA was done in accordance with the protocol set by the manufacturer’s instructions. This project was approved by the University of Pittsburgh Institutional Review Board (IRB # 060991).

From January 2015 to January 2016, data from 2164 periodontal patients were analyzed. Of this, 227 patients fit the initial criteria of having at least ten teeth and more than one periodontal evaluation. All these subjects had a diagnosis of moderate to severe periodontitis and a code D1110 in their records that confirmed oral hygiene instructions were provided. No individuals selected for our study had any indication that they had bleeding disorders, physical disabilities that would affect oral hygiene ability or were pregnant. The intervention of interest, oral hygiene instructions, is provided to patients by all students at the same time at the initial appointment. Students are trained to present patients with the same content. We used the record that oral hygiene instructions were done and that patients were charged as the confirmation the intervention was provided. The number of marked surfaces for each tooth was recorded for both plaque and bleeding indexes (Löe and Silness, [4]; Silness and Löe, [5]) of each eligible patient. We determined full mouth percentages for affected areas, as well as by quadrant, left/right side, maxillary/mandibular, anterior/posterior, and incisor, canine, premolar, and molar teeth locations. From this, we sought to identify patterns to ascertain changes of the indexes over time. Following the analysis of clinical information, participants were grouped based on the number of evaluations; 104 selected subjects received only two periodontal evaluations over time (one every 6 months to 1 year) and 123 subjects received multiple periodontal evaluations ranging from three to eight visits (one evaluation every 6 months to 1 year). Several subgroup categories (based on if oral hygiene improved) were utilized to further determine trends in the oral hygiene surrogates we analyzed (plaque and bleeding indexes) including age, ethnicity, sex, tobacco use, and other concomitant health concerns/medication use such as diabetes, cardiovascular diseases, cancer, and gastrointestinal conditions. Of the total study population, 54% were aged 50 and above, 71% identified as White, and 21% as Black. In addition, 52% of study subjects identified as being female, 69% were determined to be non-smokers, and 46 out of 227 individuals were reported to have diabetes.

Full mouth plaque indexes ranged from only 9.4% of the mouth affected to 100% (mean 47%). This variation was not statistically significant when sex was considered. When Blacks and Whites were compared, Black individuals had more teeth bleeding than White individuals (*p* < 0.05) (Table 1). The caries experience of the cohort was very high, with mean DMFT (Decayed, Missing due to caries, Filled Teeth) 15.62 (ranging from 0 to 28) and mean DMFS (Decayed, Missing due to caries, Filled Surface) 52.55 (ranging from 0 to 128). Only six individuals were caries free and 14 total individuals had a DMFT score of 2 or lower.

**Table 1:** Full mouth plaque and bleeding indexes means based on sex and ethnic background

Indexes	Relative frequency of full mouth affection
Total sample (*N* = 227)	
Plaque: Mean (Minimum-Maximum)	49 (9.4–100)
Bleeding: Mean (Minimum-Maximum)	25.2 (0.6–95)
Males	
Plaque: Mean (Minimum-Maximum)	50.1 (11.2–100)
Bleeding: Mean (Minimum-Maximum)	25.6 (0.6–95)
Females	
Plaque: Mean (Minimum-Maximum)	47.9 (9.4–100)
Bleeding: Mean (Minimum-Maximum)	24.8 (1.5–90.5)
Black	
Plaque: Mean (Minimum-Maximum)	50.1 (9.4–99)
Bleeding: Mean (Minimum-Maximum)	34 (9.4–92.5)
White	
Plaque: Mean (Minimum-Maximum)	48 (10.7–100)
Bleeding: Mean (Minimum-Maximum)	19.7 (0.6–95)

### Genotyping

Genomic DNA was isolated from saliva samples according to established protocol. Taqman chemistry was used to determine genotypes for interleukin-1 alpha (*IL-1α*) and interleukin-1 beta (*IL-1β*) markers. These interleukins act as pro-inflammatory cytokines in the immune response towards infections and have been suggested as markers for periodontitis risk (reviewed in [6]). Reaction mixes were performed in 4-μL volumes using the two markers rs1800587 for *IL-1 α* and rs1143634 for *IL-1 β*. A non-template control (using water instead of DNA) was used as a negative control to ensure quality control of genotyping reactions. Clinic, demographic, and genotyping raw data can be seen in the Additional file 1.

### Statistical analysis

Following subject genotyping, chi-square or Fisher’s exact tests were used to determine Hardy-Weinberg equilibrium and to assess the significance of the differences observed in patterns of oral hygiene identified as well as genotypic and allelic frequencies between oral hygiene patterns as implemented in the PLINK software [7]. The distributions of subjects for each interleukin genotype were compared based on four out of the initial seven aforementioned subgroups found to be statistically significance. These included sex, ethnicity, tobacco use, and diabetes status, and were ultimately used in the study as key variables in determining evaluation trends. Intervention outcomes (improvement and worsening for subjects with data from only two assessments; or improved, worse, or fluctuation of oral hygiene surrogates over time for subjects with multiple assessments) provided an additional layer of characterization for this study population. We used chi-square and Fisher’s exact tests for all comparisons. When considering oral hygiene status after the intervention based on sex, ethnic background, tobacco use, diabetes status, and interleukin genotypes, we applied Cochran-Mantel-Haenszel and regression models as implemented in the PLINK software [7]. A *p*-value of 0.05 or less was considered to be of statistical significance.

## Results

In the first phase of statistical analysis (data not shown), separation of study subjects by interleukin genotypes revealed that genotypic and allelic differences in four of the seven subgroups were in fact borderline significant or significant. These included sex (*p* = 0.07) and ethnicity (*p* = 0.02) for *IL-1 β*, and tobacco use (p = 0.07) and diabetes status (*p* = 0.06) for *IL-1 α*. In terms of allelic differences, females were more likely to have the A allele and males were more likely to have the G allele. Additionally, for *IL-1 β*, White individuals were more likely to have the G allele when compared to Black individuals and those of other ethnicities. Further exploration of these data confirmed that this statistically significant difference was the result of population substructure, with those originating from Africa to be more likely to have the G allele for this interleukin. For *IL-1 α*, diabetics tended to have the G allele more frequently than those without diabetes and the A allele tended to be more prevalent for smokers than non-smokers. These initial findings suggested that further analysis needed to be done within these subgroups in terms of intervention outcome. The assigning of intervention outcomes (improved, worsened, or fluctuated) was based on plaque and bleeding indexes calculated for each visit and compared over time. Those who fluctuated did not display any clear trend of overall improvement or overall worsening in oral hygiene indexes. Only seven subjects did not show any changes (they stayed the same) and were not included for further analysis. The variation in number of available oral hygiene assessments was due to recording the oral hygiene, extension of follow-ups, and patient compliance to follow-up visits.

Results from comparisons between intervention outcomes within subgroups and interleukin genotypes are summarized in Table 2. Ethnicity was found to be the only subanalysis with statistical significant difference in that individuals not defined as African Americans or Whites were more likely to be inconsistent and fluctuate during treatment (*p* = 0.02). Also, 99 out of 148 White individuals showed an inconsistent pattern of oral hygiene status in comparison to 19 out of 56 African Americans (67% vs 34%; *p* = 0.0002). Sex, tobacco use, and diabetes status differences were not found to be statistically significant different for intervention outcome, although we can see a trend for non-smokers to not improve and diabetics that fluctuated more often improving by the end of the observation period.

**Table 2:** Frequencies and Statistical Analysis of Intervention Outcomes for Plaque and Bleeding Indexes

	Improved (*N* = 109)	Worsened (*N* = 118)	*p*-value*	Fluctuated (*N* = 123)	Fluctuated Improved (*N* = 73)	Fluctuated Worsened (*N* = 50)	*p*-value**
Males	54	50	0.27	62	38	24	0.66
Females	55	68		61	35	26	
White	74	74	0.66	99	61	38	0.02
Black	26	30		19	12	7	
Other	9	14		5	0	5	
Smoker	35	25	0.06	41	28	13	0.15
Non-Smoker	74	93		82	45	37	
Diabetes	10	11	1.0	15	12	3	0.08
No Diabetes	99	107		108	61	47	
Genotype							
*IL-1 α*							
AA	20	18	0.23	16	10	6	0.79
AG	18	27		26	14	12	
GG	14	10		11	7	4	
*IL-1 β*							
AA	3	6	0.55	4	3	1	0.78
GA	18	16		23	13	10	
GG	36	41		43	26	17	

*p-value calculated using Chi-square test compares individuals whose plaque and bleeding indexes improved or worsened over the course of treatment. **p-value calculated using Chi-square test compares individuals whose plaque and bleeding indexes fluctuated but improved or fluctuated but worsened over the course of treatment. *p* ≤ 0.05 were considered to be statistically significant

**Fig. 1:**
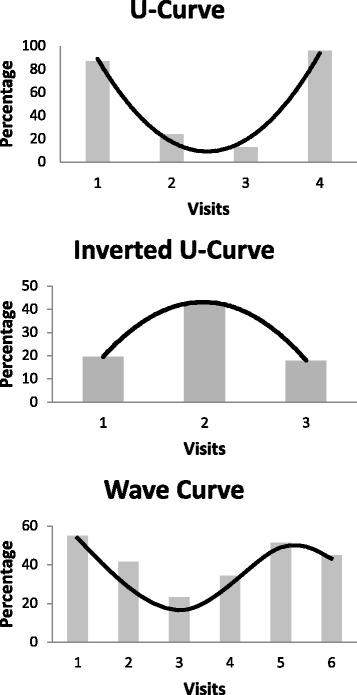
Sample graphical representations of trend line patterns for individuals with more than two oral hygiene assessments (*N* = 100) whose plaque and bleeding index percentages fluctuated throughout periodontal treatment. Statistical results and number of subjects in each fluctuation subgroup are recorded in Table 2

Of particular interest during the study was to better understand the trends present for study subjects whose progress fluctuated throughout treatment. Individuals in this subpopulation were further separated based on if their plaque/bleeding indexes improved or worsened overall from their first periodontal evaluation to their last evaluation. Data for each subject was charted and graphed and from this, three progress curve patterns were determined as depicted in Fig. 1. A U-curve trend line defined subjects with initially high plaque/bleeding indexes that decreased and improved over time but increased and returned to high-percentages nearing the end of recorded treatment. An inverted U-curve trend line was also seen, that displays initially lower plaque/bleeding indexes that increased and worsened over time but ultimately dropped again to lower levels towards the end of their last recorded evaluation. The third pattern had a wavy curve. This pattern encompasses study subjects with a combination of both U- and inverted U-curve trend lines. With these definitions in mind, these curve patterns were compared against the four significant subgroups and interleukin genotypes to identify statistically significant differences between each if in fact present (Table 3). Previous caries experience determined based on DMFT/DMFS scores did not show any differences in the distribution of the assigned patterns of improvement or worsening of the plaque/bleeding indexes (i.e. among the 14 individuals with DMFT scores up to 2, six got worse overtime and eight got better; this trend could be seen throughout in the data).

**Table 3:** Progress Curve Pattern Distributions and Statistical Analysis of Subjects with Fluctuating Intervention Outcomes

	U-Curve (*N* = 16)	Inverted U-Curve (*N* = 19)	Wavy Curve (*N* = 13)	*p*-value*
Males	4	14	7	0.0012
Females	12	5	6	
Caucasian	13	13	12	0.066
African American	3	4	1	
Other	0	2	0	
Smoker	7	4	6	0.018
Non-Smoker	9	15	7	
Diabetes	1	2	4	0.027
No Diabetes	15	17	9	
Genotype				
IL-1 α**				Genotype
AA	4	5	6	*p* = 0.3
AG	10	10	3	Allele
GG	2	4	4	*p* = 0.93
IL-1 β**				Genotype
AA	1	1	1	*p* = 0.96
GA	4	6	3	Allele
GG	11	12	9	*p* = 1.0

*p-value calculated using Fisher’s exact test compares the progress curve patterns of subjects with fluctuating intervention outcomes

**p-values for genotypes were calculated for each interleukin based on genotype and allele ratios

*p* ≤ 0.05 were considered to be statistically significant

Cochran-Mantel-Haenszel test or regression did not show different results and are not presented here for simplicity

In the fluctuation subpopulation, sex, tobacco use, diabetes status, and interleukin genotype were found to be statistically significant different in this group although not initially observed in the total sample. In regards to sex, more females presented with evaluation percentages defined by the U-curve pattern (*p* = 0.001). In contrast, more males displayed trends representative of the inverted U-curve pattern. For tobacco use, non-smokers more frequently displayed an inverted U-curve pattern (*p* = 0.02). Additionally, non-diabetics more frequently displayed a U- or inverted U-curve progression pattern whereas more diabetics were likely to follow a wavy curve pattern (*p* = 0.03).

## Discussion

Oral hygiene instruction is an intervention that is provided essentially to every patient that visits a dental office, at least on the initial visit. There are thousands of scientific papers published on the topic of dental health education (reviewed in [3, 8, 9]) and the overall conclusion continues to be that there is weak evidence that improvements in knowledge lead to improved oral health behavior, at least in the short-term. The evidence is stronger for improving oral hygiene and gingival health by using psychological behavior change models [individually tailored oral health approaches, motivational interviews, autonomy-supportive interviews, counseling with six-step method, oral hygiene education based on social cognitive aid implementation theory, transtheoretical behavior change counseling [3]] but these models are not routinely used in a dental clinic such as the one we have in our school. Also, the dentists do not certify or assess if the patients understood the oral hygiene instructions received.

Since we have longitudinal data available, we decided to explore the presence of patterns that can be clinically useful. When the sample is analyzed as a whole, our data show no obvious changes in oral hygiene practices after treatment is initiated and an oral hygiene intervention is provided as measured by presence of dental biofilm and/or gingival bleeding. When these indexes were evaluated over time in patients receiving treatment for periodontitis, we noticed at least three clinical patterns: some patients obviously improved, some patients stayed the same or even got worse, and some patients fluctuated. Among the ones who fluctuated, some showed worsening that eventually returned to levels similar to baseline, some showed improvement that eventually returned to levels similar to baseline, and some continued to fluctuate. We believe these patterns need to be accounted for in future evaluations on the impact of any intervention (educational, clinical, or biological) aiming to improve oral health. Also, it is important to highlight that this study did not aim to compare subjects based on their periodontal disease classification, although most of the subjects had moderate to severe periodontitis, and we cannot draw any conclusions on the effects of these data on periodontal disease per se. Other limitations of our data are due to the inherited nature of our design. We relied on records that were filled by a number of different professionals in training. Although, we expect a good level of consistency, data are potentially affected by differences in how information is recorded or if information is missing. The evaluation of periodontal patients in particular and subjects in general with poorer oral health indicators may limit the potential of our results to be generalizable to other populations or geographic locations.

A randomized clinical trial is probably the ideal study design to demonstrate how effective is oral hygiene instructions provided at the dental office. However, due to its cost, this is an unlikely option for future studies. We approached the problem by using a pragmatic design, meaning that the data we studied came from a clinical setting where patients are treated routinely and not under controlled conditions. This approach brings the obvious challenges of understanding the multitude of influences that impact an individual response to oral hygiene instructions. Despite these characteristics, it is remarkable to see that oral hygiene quality, as measured by presence of plaque and gingival bleeding, have different patterns depending on the individual. One would assume that patients under dental treatment would be motivated to keep excellent oral hygiene levels but our data showed that individuals will not necessarily improve, which would suggest that professional oral hygiene interventions more often than twice a year might be warranted to certain individuals.

There is plenty of evidence that show that the success of oral health promotion interventions delivered in the dental office depends on the professional person’s character, values, personality, and people skills. Data show that if professionals do not believe oral hygiene instructions will improve the health of their patient, then that professional will be less likely to practice an effective oral health promotion strategy [3]. In our sample, it was not possible to control for the professional delivering of oral hygiene instructions or the way that oral hygiene instructions were delivered (timing during consultation, length of time, how information was presented). Either a third or fourth year dental student provides oral hygiene instructions to our patients and in general they are very enthusiastic and passionate. But obviously, even among the students, there are more or less extraverted individuals, effective communicators, and proficient speakers. There was also no way to control for our patients’ previous experiences. All of our patients have certainly gone to other dentists in the past and they received oral hygiene instructions before done by others, including exposure to oral health information in school and through television adds. The only assumption we can make is that despite these past experiences, the individuals that comprised our sample still needed periodontal treatment.

Genotypes for interleukin 1 have been suggested as potential markers that could help identify individuals that may have higher risk for tooth loss [10, 11]. By extension, this genomic assessment could help determine which individuals could visit the dentist less often since they would have decreased risk of tooth loss and would not benefit from additional preventive visits [10]. This suggestion, however, was refuted by later analyses [12]. We have suggested that looking at the combination of smoking, diabetes, cardiovascular diseases, and interleukin 1 genotypes may help determine who will benefit from coming more often to the dentist [11]. Our data showed that the interleukin 1 genotypes did not associate with the longitudinal patterns of oral hygiene practice surrogates that we studied. Interestingly, the “fluctuating” pattern was found more often in Whites comprising the studied sample. Specific associations with sex, diabetes status, and smoking were also found. We believe these results indicate the possibility that biological factors (i.e. genetic variants) related to behavior may be underlying these associations. Self-motivation to diet and exercise may be dictated by the same genetic variants influencing oral hygiene compliance. Similar arguments can be made for genetic variants that determine the will to quit smoking. The data also support differences between sexes.

Pittsburgh is the largest city adjacent to one of the poorest areas in the USA, Appalachia. This region is known for its predominantly settling of Anglo-Scottish people and poverty and subsistence living that has permeated the social and cultural structure of the region. Quantitative data clearly show socioeconomic indicators are much worse for the communities in the Appalachian region compared to those in the rest of the United States (reviewed in [13]). In regard to health indicators, Pittsburgh reflects what is found in the Appalachian region, and the population treated at the University of Pittsburgh Medical Center has some of the worst health indicators in the country, which makes Pittsburgh a perfect laboratory for studying disease risks. Despite these similar characteristics, our data showed that patients would respond differently to an intervention such as oral hygiene instructions. That makes us believe that the psychology of behavior change is potentially one of the key factors (if not the key factor) to oral health promotion. It is apparent that teaching health psychology to dentists would make oral health promotion more effective in dentistry [3].

## Conclusions

Our data showed that response to interventions such as oral hygiene instructions have multiple identifiable patterns that may impact success and longevity of dental treatments.

## Additional file

Additional file 1: Clinical, demographic, and genotyping data used in this study. (XLSX 49 kb)

## Abbreviations

DMFS: Decayed, missing due to caries, filled surfaces
DMFT: Decayed, missing due to caries, filled teeth
DNA: Deoxyribonucleic acid
*IL-1 α*: Interleukin 1 alpha
*IL-1 β*: Interleukin 1 beta
IRB: Institutional Review Board

## References

[1] Yee R, Sheiham A (2002). The burden of restorative dental treatment for children in third world countries. Int Dent J.

[2] Pitts N (2004). Are we ready to move to non-operative/ preventive treatment in dental caries in clinical practice?. Caries Res.

[3] Kay E, Vascott D, Hocking A (2016). A review of approaches for dental practice teams for promoting oral health. Community Dent Oral Epidemiol.

[4] Löe H, Silness J (1963). Periodontal disease in pregnancy. I. Prevalence and severity. Acta Odont Scand.

[5] Silness P, Löe H (1964). Periodontal disease in pregnancy II. Acta Odontol Scand.

[6] Grigoriadou ME, Kputayas SO, Madianos PN, Strub JR (2010). Interleukin-1 as a genetic marker for periodontitis: review of the literature. Quintessense Int.

[7] Purcell S, Neale B, Todd-Brown K, Thomas L, Ferreira MA, Bender D, Maller J, Sklar P, de Bakker PI, Daly MJ, Sham PC (2007). PLINK: a tool set for whole-genome association and population-based linkage analyses. Am J Hum Genet.

[8] Brown L (1994). Research in dental health education and health promotion: a review of the literature. Health Educ Q.

[9] Kay L, Locker D (1996). Is dental health education effective? A systematic review of current evidence. Community Dent Oral Epidemiol.

[10] Giannobile WV, Braun TM, Caplis AK, Doucette-Stamm L, Duff GW, Kornman KS (2013). Patient stratification for preventive care in dentistry. J Dent Res.

[11] Vieira AR, Hilands KM, Braun TW (2015). Saving more teeth – a case for personalized care. J Pers Med.

[12] Diehl SR, Kuo F, Hart TC (2015). Interleukin 1 genetic tests provide no support for reduction of preventive dental care. J Am Dent Assoc.

[13] McGarvey EL, Leon-Verdin M, Killos LF (2011). Health disparities between Appalachian and non-Appalachian counties in Virginia USA. J Community Health.

